# School-based peer education intervention on physical activity in Iranian adolescent girls: an application of the theory of planned behavior

**DOI:** 10.3389/fpubh.2025.1558210

**Published:** 2025-05-07

**Authors:** Afsaneh Ranaei, Seyedeh Belin Tavakoly Sany, Mohammad Vahedian Shahroodi, Azam Sabahi, Hadi Tehrani, Abdoljavad Khajavi

**Affiliations:** ^1^School of Health, Student Research Committee, Mashhad University of Medical Sciences, Mashhad, Iran; ^2^Department of Health, Safety, Environment, School of Health, Mashhad University of Medical Sciences, Mashhad, Iran; ^3^Social Determinants of Health Research Center, Mashhad University of Medical Sciences, Mashhad, Iran; ^4^Department of Health Education and Health Promotion, Faculty of Health, Mashhad University of Medical Sciences, Mashhad, Iran; ^5^Department of Health Information Technology, Ferdows Faculty of Medical Sciences, Birjand University of Medical Sciences, Birjand, Iran; ^6^Department of Family and Community Medicine, School of Medicine, Gonabad University of Medical Sciences, Gonabad, Iran

**Keywords:** physical activity, peer education, health education, health promotion, public health

## Abstract

**Introduction:**

The level of physical activity (PA) among Iranian adolescent girls is significantly lower than the recommended level. This survey aims to examine the impact of school-based peer education Intervention on promoting PA using the theory of planned behavior (TPB) among girls in high school.

**Methods:**

A quasi-experimental study was conducted on 160 girls in high school of Gonabad, Iran. School-based peer education program was conducted for the intervention group through five training sessions and the three-month follow-up to enhance clarity. Data was collected using a researcher-made questionnaire based on TPB, the International Physical Activity Questionnaire (IPAQ), and demographic information. The content validity Index, content validity ratio, and Cronbach’s alpha coefficient values demonstrated strong content validity and reliability (> 0.85). The obtained data were analyzed using appropriate statistical tests using SPSS software 20.

**Results:**

The average age of the participants was 13.5 ± 1.2 years, and 65% had low levels of PA. The intervention group demonstrated significant improvements across all constructs of the TPB compared to the control group. The mean scores for attitude toward behavior (*p* = 0.012), subjective norms (*p* = 0.023), perceived behavioral control (*p* = 0.003), and behavioral intention (*p* = 0.001) showed significant enhancement in the intervention group. Additionally, physical activity behavior improved significantly (*p* < 0.05) after the intervention, while no significant improvement was observed in the control group (*p* > 0.05). The Cohen’s d values for all constructs in the intervention group, except for subjective norms, exceeded 0.8, indicating a large effect size for these constructs.

**Conclusion:**

School-based peer education using the TPB can be used as an effective theory to promote the level of PA among high school girls. This approach strengthens attitudes, perceived behavioral control, and subjective norms, leading to improved behavioral intentions and increased PA among female students.

## Background

Insufficient physical activity (PA) is the most significant global risk for mortality (1) and has a crucial role in maintaining and modifying mental and physical health, particularly among adolescents (2, 3). Physical activity can improve fitness, reduce stress, boost self-esteem, and prevent chronic diseases (4, 5). World Health Organization (WHO) reported that globally, 81% of adolescents aged 11–17 are not sufficiently active for ≥60 min/day during 7 days, and the level of PA decreases by as much as 7% per year among adolescents (6, 7).

Studies have indicated that the level of PA of boys and girls’ students differs, with the decline becoming steeper and starting sooner among girls. Some evidence supports that gender significantly influences intentions and behaviors related to regular PA (8). A recent review on girls indicated that school-based PA programs had only a small effect on the level of PA and health indicators (9). Therefore, improving PA among adolescent girls is a public health priority, and needs novel intervention programs to increase PA. Internal Factors influencing girls’ participation in PA include psychological correlates (attitude, enjoyment of PA, self-efficacy, and perceived competence, as well as external factors, including ‘sporty’ stereotypes, friendship quality, competing priorities, and peer and family support and affiliation) (10).

One effective approach to enhancing PA among adolescents is peer education because it is a key element of the social network that transitions children from childhood to adulthood. Peers can act as role models and sources of social support because adolescents are less dependent on their families in this transition and have closer relationships with their peers who give them the motivation and attitude necessary for any change in their behavior and performance (11–13). Understanding the social and psychological dynamics of adolescents is essential for recognizing why peer education is a key tool for improving health behaviors and outcomes. Key dynamics such as emotional development, peer influence, identity formation, and cognitive development allow peer education to effectively address complex health and social issues. This approach enhances adolescents’ decision-making abilities and critical thinking skills, ultimately leading to better outcomes (14–16). Watson’s review, which assessed the impact of classroom-based PA interventions on academic and PA outcomes, showed that such interventions could positively influence students’ academic performance and PA (14, 15). Likewise, several studies support that school-based peer education interventions can significantly reduce sedentary behavior and modify the level of health behaviors (fruit intake, eating disorders, and use of drugs and alcohol) in school students. Peer-based interventions via social models and theories could be practical to help adolescent girls become more physically active (17, 18).

In past decades, many psychosocial theories such as the Theory of Planned Behavior (TPB) were effectively applied to understand psychosocial determinants of PA behaviors among adolescents (19). The TPB is a successful cognitive framework for comprehending and predicting changes in health behavior and is widely used in studies on PA in different populations (e.g., elementary school students, obese students, older adults and patients with chronic diseases) (20, 21). This theory posits that the closest determinant of behavior is valuable to predict key elements that affect an individual’s intention to perform the health behavior, which is shaped by motivation and willingness to take action (19). Several studies indicated a robust and significant association between the PA behaviors and constructs of the TPB among adolescent girls, which is able to predict for 24–36% of the variation in behavioral intentions (22). Behavioral intention is influenced by three main constructs: the individual’s attitude towards the health behavior (negative or positive assessment of behavior, e.g., regular PA is tiring and boring but also fun and refreshing), subjective norms (social pressure or perceived expectations from key peoples like parents, family members, and friends), and perceived behavioral control (degree of individual’s confidence in their capacity to control the health behavior) influence behavioral intention and actual participation in health behaviors such as PA (23, 24). Identifying predictors of behavioral intention is essential for promoting PA intentions and behaviors and aids in designing effective educational interventions. Numerous studies have shown a strong correlation between TPB constructs and PA behavior, and sports research has supported TPB’s predictive capability for exercise behavior (23, 25–27).

In Iran, studies have shown that approximately 70% of adolescents do not reach the recommended level of PA, with girls being significantly less active than boys (4, 26). This can be due to social and cultural norms (dress codes, limited access to gender-segregated facilities, prioritization of work/study), urban infrastructural (lack of safe spaces and air pollution), and economic barriers (cost of Gym memberships and uneven resource distribution) (28). Likewise, evidence shows that weak physical education programs in schools and social media about the benefits of PA and modern lifestyle shifts, including sedentary Jobs (reliance on vehicles and desk-bound work) and Screen Time (use of TVs and smartphones for hobbies) can hinder PA among girls and reduce their participation in regular PA. Furthermore, political factors in recent decades have led to a limit on funding to develop sports infrastructure and public health facilities (29). As far as our review has shown, no study in Iran has yet examined the impact of school peer-education interventions based on the TPB on improving PA among female students. Only a few studies have evaluated the effect of a school-based peer education on PA in adolescents in Iran. These studies used a teacher-led intervention to improve PA. It is still not clear what type of attitude and subjective norms control behavioral intention and actual participation in PA among Iranian girls (24, 30). Given that peer education and the Theory of Planned Behavior offer a novel approach to promoting PA, which can have long-term benefits for physical and mental health, this study aims to address these gaps and contribute to the existing knowledge. Therefore, the primary objective of this study is to examine the impact of a peer educational intervention on promoting PA among high school girls using the TPB.

## Methods

### Study design, setting, participants and sampling

This study was conducted on 160 high school girls from two public schools in the city of Gonabad, Iran. This is a quasi-experimental with two parallel arms (the intervention and control groups) consisted of 80 participants (a total of 160 students). The sample size was calculated using the sample size formula based on the average of two independent groups, according to the values obtained in similar studies by Salhi et al. (23), with a confidence limit of 95% and a test power of 90% for each group. The type II error (*β*), type I error (*α*), and confidence coefficient (*Z*) were 20%, 0.1, and 1.64, respectively. The estimated sample size was 80, considering an attrition rate of 10% for each group (Equation 1).


(1) $$n=\frac{2\times \left(Z\alpha /2+Z\beta \right)2\cdot \left(\sigma ₁2+\sigma ₂2\right)}{\left(M₁-M₂\right)2}$$

**Figure 1:**
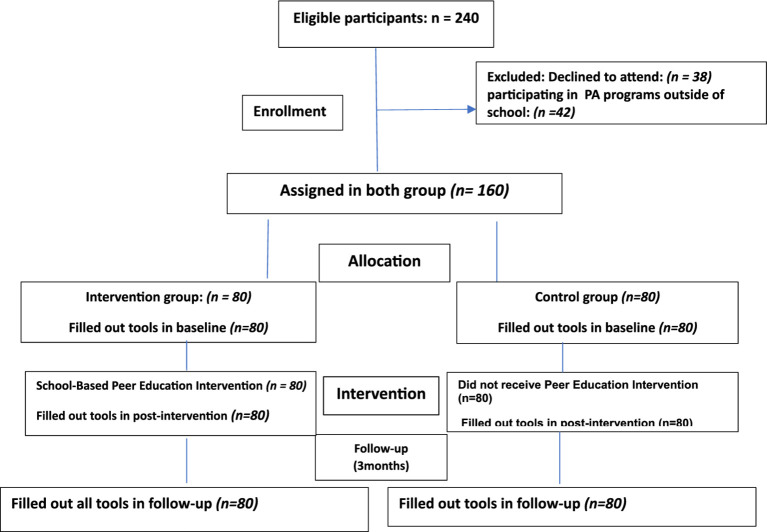
Flow of participants through each stage of the intervention program.

For sampling, a list of all-girl high schools in the city of Gonabad was obtained, and 2 public girls high schools were randomly assigned to control (*n* = 1) and intervention (*n* = 1) groups. The target population for this study included high school girls aged 14–18 years from two different public schools in Iran. These two schools were specifically selected from areas that had no prior connections with each other to ensure that the intervention and control groups remained completely independent, with no contact between participants of the different groups before the start of the study. After estimating the target sample size, 160 students who met inclusion criteria were randomly chosen from each school to participate in intervention (*n* = 80) and control (*n* = 80) groups (Figure 1).

Students entered the study who were satisfied with the study, their age was between 14 and 18 years and had no history of chronic illness or physical problem. The exclusion criteria included students who were unwilling to continue their studies, those already participating in organized PA programs outside of school, or individuals who developed health issues during the study that could impact their ability to engage in the required physical activities. Additionally, students who were unable to complete the post-test and follow-up questionnaire were also excluded from the study.

### Outcome assessment

The primary objective of this research was to modify PA behaviors based on the TPB. The TPB-based intervention and demographic characteristics served as the independent variable and covariates in this study, respectively. The questionnaire was divided into three sections. The first section focused on collecting demographic information, including age, gender, parental education, and parental occupation. The second section was designed according to constructs of the TPB and included four main constructs with a total of 23 items.

To develop the TPB tools, we reviewed relevant literature on the TPB and conducted interviews with 30 students to gain insights into their opinions regarding PA behaviors (31). The questionnaire was reviewed by several experts in the field of health and health behavior to assess the relevance and necessity of all questions and ensure that all items had adequate content validity. The Content Validity Index (CVI = 0.92) and Content Validity Ratio (CVR = 0.87) both demonstrated strong content validity (above 0.85). The questionnaire was pilot tested on a small sample of the target population, and its internal consistency was assessed using Cronbach’s alpha. The results showed acceptable reliability, with Cronbach’s alpha coefficient values of 0.79, 0.89, 0.88, and 0.90 for Attitudes, Subjective Norms, Perceived Behavioral Control, and Behavioral Intention, respectively.

Attitudes is the first construct of TPB, and it included 8 items that assessed the participants’ attitudes towards PA (e.g., how beneficial do you believe regular PA is for your health? And how enjoyable do you find engaging in regular PA?) with five-point Likert ranging from 1 (strongly disagree) to 5 (strongly agree). The score for this construct ranged from 8 to 40.

The subjective norms construct included six items that assessed social pressures and perceptions related to PA behavior, specifically from friends and classmates. For example, questions included, “To what extent do you think your family and friends support you in doing regular PA?” and “To what extent do your classmates encourage you to participate in PA?” Respondents rated their answers on a five-point Likert scale for rating. The scores for this construct ranged from 6 to 30.

The perceived behavioral control construct included five items that assessed participants’ perceptions of barriers (e.g., “How difficult do you find it to engage in regular PA when you have a busy schedule?” and “To what extent does lack of access to sports facilities prevent you from being PA?”) and facilitators (e.g., “How confident are you that you can maintain regular PA despite feeling tired?” and “How likely are you to engage in PA if you have the support of your peers?”). Responses were measured on a five-point Likert scale. The scores for this construct ranged from 5 to 25.

The Behavioral Intention construct comprised four items designed to assess participants’ intentions to engage in PA in the future. For example, participants were asked, “To what extent do you intend to exercise regularly in the coming months?” and “How likely are you to participate in PA at least three times a week in the near future?” Responses were measured using a five-point Likert scale. The total scores for this construct ranged from a minimum of 4 to a maximum of 20.

The third section of the questionnaire included items from the short version of the standardized International PA Questionnaire (IPAQ) (3). First, all participants were guided through the questionnaire, and we evaluated their PA levels and weekly caloric expenditures. Types of physical activities were classified into five categories: vigorous, moderate, walking, sitting, with their standard MET values being 8, 4, 3, 3, and 1, respectively. The total MET for each type of physical activity was calculated by multiplying the corresponding MET values by the duration of physical activities (in minutes). The IPAQ categories are based on MET-min/week: low (<600), moderate (600–3,000), and high (>3,000) (4). The validity and reliability of the IPAQ have been tested in Iran, making it a standard tool for accurately measuring PA levels (11, 12, 26).

### Intervention

The primary objective of the intervention was to promote PA among adolescent girls using peer education based on TPB. This intervention aimed to improve participants’ attitudes, subjective norms, perceived behavioral control, and behavioral intentions regarding regular PA (Table 1). The rationale for selecting five educational sessions over a five-week period varies depending on the target population, health behavior objectives, and key constructs of the Theory of Planned Behavior (TPB). Furthermore, several studies have supported the idea that conducting four to six sessions within a two to six-week timeframe can positively impact the promotion of healthy behaviors through TPB-based training. This duration provides sufficient time for facilitating participant skill development and engagement (32, 33).

**Table 1:** Intervention based on the TIDIER checklist.

Tidier item	Description
Brief name	Peer Education Program for improving PA.
Why	The impact of school peer-education interventions based on the TPB on improving PA among female students has not been studied yet. It is still not clear what type of attitude and subjective norms control behavioral intention and actual participation in PA among Iranian.
What	Manuals - Brochures - Educational videos.
What (Procedures)	School-based peer education was conducted in the intervention group based on five-week sessions with a focus on the TPB. The peer training program did not include a control group. The control group did not receive any training and attended a different school than the intervention group.
Who provided	Specialist in exercise specialist and health trainer, health education and promotion.
Where	Classrooms in secondary schools located in Ferdows city, South Khorasan province - Number of schools: 2 (1 intervention group, 1 control group).
When and how much	5 sessions for participants by peer group- Each session lasts 2 h - Held on specific days of the week.
Tailoring	The intervention is adapted based on the specific needs of different groups.
Modifications	No modifications occurred.
How well (Planned)	Adherence to the protocol intervention was optimal for all participants and all participants attend in follow-up.
How well (Actual)	The complete (100%) scheduled intervention program was delivered. The implementation of the intervention according to the planning was reviewed with regular monitoring and evaluation. Adherence to the program was high, and sessions were conducted as scheduled. Changes were made only as needed, and reports indicated that the intervention was generally carried out in accordance with the original plan.

The sessions were designed to be interactive and focused on the constructs of TPB to fully engage the participants (Table 2). Additionally, support was provided through phone contact and internet messaging services to encourage students to be more active. This educational intervention was delivered by peers under the supervision and management of an exercise specialist and a health trainer during the fifth session. In this educational program, each session focused on one of the constructs of TBP to comprehensively prepare participants for engaging in PA.

**Table 2:** Educational content and activities conducted in intervention sessions based on the theory of planned behavior.

Session	TPB constructs	Intervention method	Intervention’s instruction	Objectives	Time
1	Attitudes	Peer education, exercise demonstrations, and sharing experiences	Peers discussed the benefits of PA, demonstrated various exercises, encouraged participants to share their personal experiences and beliefs.	Educate about the benefits of PA, demonstrate different exercises, increase awareness, and create a positive attitude towards PA.	60 min
2	Subjective norms	Group discussions and role-playing led by peers	The impact of peers, family, and community on PA behaviors was examined through group discussions and role-playing, and positive social influences were reinforced.	Identify and strengthen positive social influences on PA, enhance social support, and increase motivation through peer and family interactions.	60 min
3	Perceived behavioral control	Discussions and goal-setting led by peers	Peers led discussions on overcoming barriers to PA and assisted participants in setting realistic and achievable goals for PA routines.	Identify and overcome barriers PA, increase self-efficacy, and set achievable goals.	60 min
4	Behavioral intention	Practical activities and group exercises led by peers	Behavioral intentions in participants were reinforced through setting specific, realistic, and achievable goals for PA.	Strengthen the intention to maintain an active lifestyle, set specific and realistic goals for PA.	60 min
5	Stabilizing PA behaviors	Progress review, feedback, and follow-up	Peers reviewed participants’ progress, provided feedback, and offered resources to support continued PA after the program. Follow-up was conducted via phone and online messaging.	Review progress, provide feedback and support, and offer resources and guidance for maintaining PA levels after the intervention.	60 min

The first session emphasized attitudes towards PA. Participants were educated about the health benefits of PA, introduced to various exercises, and encouraged to share their personal experiences and beliefs. This approach helped create a supportive learning environment. The second session focused on subjective norms. It explored the influence of peers, family, and society on PA behaviors. Group discussions and role-playing exercises were conducted to help participants recognize and reinforce positive social influences on their decisions regarding PA. The third session centered on perceived behavioral control. Participants identified barriers and facilitators to PA and explored strategies to overcome these barriers, thereby enhancing their sense of control over their PA behavior.

The fourth session emphasized behavioral intention. Participants were guided to set specific, realistic, and achievable goals for their PA, reinforcing their intention to maintain an active lifestyle. Finally, the fifth session was dedicated to reinforcing and practicing the concepts covered in the previous sessions. Participants engaged in practical activities and group exercises to address any remaining challenges. They also received feedback and were provided with resources to support their continuation of PA after the program ended. This step-by-step structure helped participants thoroughly understand the key concepts and effectively achieve the desired behaviors.

To ensure ongoing support, participants were followed through telephone and internet messaging services, providing encouragement and personalized guidance. Educational materials, such as pamphlets, booklets on PA, visual aids like posters and videos, and goal-setting worksheets, were utilized during the sessions. Participants who achieved their PA goals received incentives, such as badges or certificates, which added a motivational component to the intervention. The sessions were conducted by trained peer educators who were chosen for their leadership skills, communication abilities, and enthusiasm for health promotion. Prior to the intervention, these peer educators participated in a two-day training workshop where they learned about the TPB, effective communication strategies, group facilitation techniques, and ways to promote PA.

Peers received training from exercise specialists, health trainers, and health educators who supervised all stages of the educational intervention from baseline to follow-up. The training workshop for peer educators utilized training manuals and engaging presentation slides to enhance their self-efficacy, awareness, and attitude toward the benefits of different exercises. Likewise, they learned how to identify and overcome barriers and set achievable goals for physical activity. Peer educators received certificates upon successful completion of the training, recognizing their achievements. Commitment and contribution.”

Throughout the intervention, regular follow-up was conducted through weekly check-ins with participants to monitor their progress and address any challenges they encountered in maintaining PA. Participants also received ongoing motivational support through text messages and phone calls. Peer educators remained available to provide advice and encouragement as needed. After the completion of the intervention, a follow-up assessment was conducted to evaluate the changes in PA levels and the impact of the intervention on the TPB constructs. This structured approach ensured that the educational sessions were engaging, informative, and effective in promoting sustained PA among adolescent girls.

This comprehensive intervention was designed not only to educate the participants but also to support them throughout their journey of incorporating PA into their daily routines. The consistent follow-up and personalized guidance ensured that the knowledge gained during the sessions translated into long-term behavior change, helping participants maintain an active lifestyle even after the conclusion of the program to ensure the accuracy of the study results, 80 participants were allocated to each group, taking into account potential dropout rates. The intervention process was carefully monitored, with continuous engagement maintained to minimize attrition. Additionally, support initiatives and motivational techniques were implemented to address challenges faced by participants, further reducing the risk of dropouts and enhancing the overall effectiveness of the program. Individuals who collected and analyzed the data were unaware of which group received the training. This blinding was conducted to avoid detection bias.

### Statistical analysis

In this study, statistical analysis was conducted using SPSS software, version 20. Descriptive statistics were employed to analyze the demographic data and summarize the descriptive statistics of PA levels. Chi-square tests, paired t-tests, and ANOVA were utilized to determine statistical differences between different groups.

compare the changes in PA levels and the constructs of the TPB among multiple groups in both groups. Likewise, Cohen’s d tested to interpret whether the significant difference between control and intervention groups is practically meaningful. Cohen’s values are commonly classified into specific effect sizes for the interpretation: large (*d* ≥ 0.8), medium (*d* = 0.5), and small (*d* = 0.2). Regression analysis assesses the strength of the relationship between a dependent variable (Physical Activity Behavior) and independent variables (TPB constructs). Correlation analysis tested the relationship between the TPB constructs and PA levels. A level of 0.05 and a 95% confidence interval were considered as the statistical significance level.

## Result

### Descriptive results

The average age of the participants was 13.5 ± 1.2 years. In terms of parental education, 101 63.13% (*n* = 101) of fathers and 62.5% (*n* = 100) of mothers were under diploma. Concerning occupation, 38.75% (*n* = 72) of fathers worked as laborers, while 76.25% (*n* = 122) of mothers were homemakers. To ensure the validity of the research findings, the baseline demographic characteristics both intervention and control groups were evaluated (Table 3). The analyses indicated that both groups were similar in all demographic characteristics, such as age, father’s education, and parental occupation, with no significant differences observed between them (*p*-value > 0.05).

**Table 3:** Participant’s demographic characteristics.

Variables *n* (%)	Categorization	Total (*n* = 160)	Control group (*n* = 80)	Intervention group (*n* = 80)	^*^Statistical test result
Age	12 years	38 (23.75)	18 (22.51)	20 (25)	0.151
	13 years	49 (30.63)	24 (30)	25 (31.25)	
	14 years	42 (26.25)	22 (27.53)	20 (25)	
	15 years	31 (19.38)	16 (20)	15 (18.75)	
Father’s education	Elementary	32 (20)	17 (21.25)	15 (18.75)	0.640
	High school	69 (43.13)	34 (42.5)	35 (43.75)	
	Associate degree	39 (24.38)	19 (23.75)	20 (25)	
	Bachelor’s or higher	20 (12.55)	10 (12.52)	10 (12.5)	
Mother’s education	Elementary	38 (23.75)	18 (22.55)	20 (25)	0.353
	High school	62 (38.75)	32 (40)	30 (37.58)	
	Associate degree	40 (25)	20 (25)	20 (25)	
	Bachelor’s or higher	20 (12.52)	10 (12.52)	10 (12.51)	
Father’s occupation	Worker	62 (38.75)	32 (40)	30 (37.55)	0.751
	Employee	49 (30.63)	24 (30)	25 (31.25)	
	Self-employed	49 (30.63)	24 (30)	25 (31.25)	
Mother’s occupation	Housewife	122 (76.25)	62 (77.51)	60 (75)	0.401
	Employee	38 (23.75)	18 (22.52)	20 (25)	

*Statistical test: Chi-square tests were used to compare demographic characteristics between groups. A significance level of 0.05 was considered. *n*, number of eligible participants.

### Comparative analyses

#### Changes in TPB constructs

The mean scores of the various constructs of the TPB in the intervention group significantly increased compared to the control group after the school-based Peer Education. In the attitude construct, the mean score for the intervention group is significantly changed from 31.07 before the training to 38.85 after the training (*p* < 0.012), while in the control, it changed from 31.93 to 31.97 (*p* = 0.175). In the subjective norms construct, the average score in the intervention group significantly increased from 21.06 to 25.63 after the training program (*p* = 0.023), whereas in the control, it changed from 21.05 to 22.33, showing no significant difference (*p* = 0.490). Regarding perceived behavioral control, the mean score in the intervention group rose from 17.21 in pre-intervention to 21.16 after the post-intervention (*p* < 0.05), while in the control group, it changed from 17.30 to 17.32, with no significant difference (*p* = 0.159). For the behavioral intention construct, the mean score in the intervention group increased from 13.78 to 17.67 after the training (*p* < 0.05), while in the control group, it changed from 14.33 to 14.37, with no significant difference (*p* = 0.083) (Table 4).

**Table 4:** Average scores for TPB constructs from baseline to follow up for physical activity in control and intervention groups.

Model’s constructs	groups	Pre-intervention	Post-intervention	*p*-value**	Cohen’s d
		Mean (SD)	Mean (SD)		
Attitude toward behavior	Control intervention	31.932 (4.185) 31.076 (3.75)	31.972 (4.334) 38.855 (4.332)	0.172 0.012	0.009 1.790
*p-value		0.172	<0.001		
Subjective norms	Control intervention	21.055 (3.73) 21.066 (3.20)	22.336 (2.991) 25.632 (4.331)	0.490 0.023	0.212 0.603
*p-value		0.982	<0.001		
Perceived behavioral control	Control intervention	17.302 (2.97) 17.211 (2.66)	17.327 (2.981) 21.164 (2.332)	0.159 0.003	0.006 1.583
*p-value		0.485	0.012		
Behavioral intention	Control intervention	14.335 (2.75) 13.782 (2.18)	14.375 (2.761) 17.672 (1.33)	0.083 0.001	0.016 1.824
*p-value		0.163	0.021		

*n*, number of eligible participants: SD, standard division; *Testing significant change between control and intervention groups; **Testing significant change from pre-intervention to Post-intervention based ANOVA; Cohen’s d effect size to assess the size of the practical difference between control and intervention groups: large (*d* ≥ 0.8), medium (*d* = 0.5), and small (*d* = 0.2).

Cohen’s d shows that the intervention group has a higher estimated value compared to the control group. In the control group, the Cohen’s d values for all constructs were below 0.2, indicating a small effect size. Conversely, in the intervention group, the Cohen’s d values for all constructs, except for subjective norm, exceeded 0.8, reflecting a large effect size for these constructs. For subjective norms, the Cohen’s d value was 0.603, representing a moderate effect size.

#### Changes in physical activity

Changes in PA levels showed that the number of individuals in the low, moderate, and high activity levels in the intervention group before the peer education program were 54, 25, and 1, respectively, and after the intervention, they increased to 22, 50, and 8 (*p* < 0.05). In the control group, the number of individuals in the low, moderate, and high activity levels before the intervention were 51, 27, and 2, respectively, and after the intervention, they changed to 48, 30, and 2, with no significant difference (*p* > 0.05) (Table 5).

**Table 5:** Physical activity levels in control and intervention groups at post-intervention.

Variables		Control group (*n* = 80)	Intervention group (*n* = 80)	*p-value
Physical activity, *n* (%)	Low	48 (60)	22 (27.5)	−0.001
	Moderate	30 (37.5)	50 (62.5)	0.001
	High	2 (2.5)	8 (10)	0.001

* Testing significant change between control and experimental groups which is significant at the 0.05 level.

Regression tests indicated that the changes observed in level of PA were significantly associated with improved attitude toward PA behavior, subjective norms, perceived behavioral control, and behavioral intention in the intervention group (Table 6). Multiple regression analysis revealed that perceived behavioral control was the strongest predictor of PA behavior among the TPB constructs. Perceived behavioral control (0.408), behavioral intention (*β* = 0.302), attitude toward behavior (*β* = 0.257), and subjective norms (*β* = 0.228) also significantly predicted PA. However, perceived behavioral control, with the highest beta coefficient of 0.40, was identified as the most influential factor in determining PA behavior. A squared value of 0.46 was recorded, indicating that 46% of the variance in PA is accounted for by the constructs of the TPB.

**Table 6:** Regression results for predicting physical activity behavior.

Variables	*B*	Unstandardized coefficient std.error	Standardized beta coefficient (*β*)	*t*-value	*p-value	VIF
Constant	12.312	4.56		2.164	0.007	
Attitude toward behavior	0.182	0.052	0.257	2.85	0.005	1.26
Subjective norms	0.172	0.034	0.228	2.50	0.013	1.12
Perceived behavioral control	0.289	0.052	0.408	4.60	0.001	1.62
Behavioral intention	0.214	0.049	0.302	3.30	0.002	1.73
R-Squared (R^2^)	0.465					

* to examine the predictive value of TPB constructs on physical activity behavior. A significant level of 0.05 was considered.

## Discussion

This study examined the impact of a school- based peer education intervention via Theory of Planned Behavior (TPB) on PA among high school girls. The findings indicated that the peer education intervention was significantly associated with improvements in PA. These results are consistent with previous studies that have confirmed the positive impact of educational interventions and the critical role of peer education in behavior change (34–36).

Based on the results, peer education could be an effective strategy for promoting behavior change, primarily due to its ability to strengthen connections and directly influence peers. The research highlighted that a school-based peer education intervention positively impacted key constructs of the TPB, particularly perceived behavioral control, attitudes toward PA, and subjective norms. Given that adolescents are heavily influenced by their peers, leveraging peers as educational resources resulted in shifts in attitudes and the reinforcement of positive social norms. These findings align with similar studies that demonstrate how peer education can lead to positive changes in health-related behaviors by fostering social interactions and creating motivating influences (35–41). Tara B. Blackshear et al., findings indicated that a school-based peer interventions program for black adolescent girls lead to enhance their self-esteem, regular PA engagement, group fitness sessions, and health outcomes (41). Likewise, Fiona McHale et al., reviewed effect of peer-led strategies on improving level of PA levels among adolescents based on the 11 high quality studies. Their review showed that appropriate peer-led strategies have a main role in providing encouragement, sharing knowledge, and promoting shift in norms and co-participation. This approach can facilitate a rotation of responsibilities and roles among the adolescents. However, a study in Pakistan found that peers did not significantly influence PA (42), which may be due to differences in cultural attitudes, societal norms, methodology, and design of studies in different communities (38).

The results of Brown’s systematic review study, aimed at examining key influences on university students’ PA, identified 56 factors that affect students’ PA, grouped into 12 categories (43). The key factors influencing PA are the environment and available resources, social influences (such as exercising with friends), and the establishment of goals (prioritizing physical activity). In contrast, less critical factors include intentions, emotions, knowledge, and skills. A review study by Silva, which aimed to identify barriers to PA among high school and university students, found that a lack of motivation is one of the primary barriers preventing students from engaging in PA (44). When students receive accurate information about the benefits of PA and observe their peers actively participating, their motivation to engage in such behaviors tends to increase. The results of this study indicate that peer education significantly improved students’ attitudes toward PA. This improvement can be attributed to the positive effect of the information shared and the support from peers, which enhanced the perceived value and appeal of PA among students. Similar to findings in previous studies, this positive impact on attitudes can lead to a change in behavior (23, 27, 45).

Analyses showed that a school-based peer education intervention may also improve subjective norms. This means that the opinions and expectations of peers can significantly influence an individual’s perception of desirable and accepted behaviors within the peer group. Stronger subjective norms regarding PA can encourage students to participate more in physical activities (25, 27, 45). A review study by Danglin Hu and colleagues, investigating the factors affecting participation in PA among school-aged children and adolescents, identified age, gender, ethnicity, and self-concept as the most prevalent individual-level factors (46). At the interpersonal and organizational levels, support from peers, parents, friends, and teachers were positive predictors of students’ participation in PA. When peers actively promote and engage in physical activity, it creates a supportive environment that normalizes and encourages such behaviors. This social reinforcement can significantly enhance students’ motivation to participate in PA, as they seek to align with the expectations and behaviors of their peer group. By improving subjective norms through peer education, interventions can effectively strengthen the culture of physical activity, leading to sustained behavior change and improved health outcomes (47).

One of the main findings of the present study is the significant association between a school-based peer education intervention and perceived behavioral control. This construct reflects students’ belief in their ability to manage and engage in physical activity, which is crucial for behavior change. Improved perceived behavioral control often leads to increased self-efficacy, as students feel more competent and confident in their ability to participate in physical activity. Peer education plays a vital role in this regard by offering practical experiences and encouragement, which enhances students’ skills and sense of control. This increased self-efficacy, in turn, motivates students to overcome barriers and persist in their PA routines. Previous research supports these findings, indicating that peer-led interventions effectively boost self-efficacy through social support and hands-on experiences, thereby enhancing students’ capability and willingness to engage in PA (23, 26, 45, 48). By improving perceived behavioral control, students are more likely to integrate PA into their daily lives, leading to sustained behavior change and healthier lifestyles. The results of this study indicate that perceived behavioral control (PBC) is a stronger predictor of health-related behaviors than behavioral intention. This supports previous studies that show a significant and direct relationship between PBC and health-related behaviors, particularly those that require specific resources or skills (managing stress or cooking healthy meals) in challenging contexts (49, 50). In communities where cultural and contextual characteristics significantly influence access to societal norms and resources, PBC plays a critical role as a determinant of health-related behaviors. Understanding this key determinant enables the development of targeted training programs that enhance PBC by fostering supportive environments and addressing specific barriers (50). In this study, the strongest relationship between perceived behavioral control and the level of PA may due to cultural norms (such as dress codes, limited access to gender-segregated facilities, and the prioritization of work or study) and contextual factors (including a lack of safe spaces, air pollution, and the cost of gym memberships) that significantly impact access to resources or skills necessary for PA (23, 24). Therefore, under these conditions, perceived behavioral control may become a critical determinant of regular PA engagement compared to behavioral intention.

Significant improvement in behavioral intention was also observed in the intervention group. This indicates that peer education successfully increased students’ motivation and intention to participate in physical activity. An increase in behavioral intention, especially when combined with improvements in other TPB constructs, can lead to positive changes in actual behaviors. These findings are consistent with previous studies that have shown the impact of TPB constructs on health-related behaviors (23, 26, 27, 45). The results of Rajeh’s study (51), which applied TPB to oral health behaviors, showed that attitudes, subjective norms, and perceived behavioral control significantly predict behavioral intentions, which in turn influence actual behaviors. Overall, behavioral intention to increase PA is very important as it reflects an individual’s motivation and commitment. According to TPB, behavioral intention is influenced by attitudes, subjective norms, and perceived behavioral control. A school-based peer education intervention can enhance these factors, thereby increasing students’ motivation and intention to be active. A strong behavioral intention predicts actual behavior and leads to increased physical activity (52, 53).

### Practical implications

This study significantly enhances the validity of the results due to the use of a strong experimental design with control and intervention groups. Additionally, the use of validated tools to assess PA and theoretical constructs increased the accuracy and reliability of the data. Another strength of this study is the use of a school-based peer education as an innovative and effective intervention strategy for promoting PA. The practical implications of this study include the development and implementation of a school-based peer education intervention based TPB to increase the level of PA among high school girls, promoting the metabolic equivalent of PA. Additionally, it is recommended that teachers and school counselors be trained in the use of peer education strategies to enhance PA, which is associated with metabolic equivalent and lead to healthier lifestyles. Policymakers and educational planners should utilize the findings of this study and leverage the potential of peer education to improve health programs and promote physical activity, ultimately enhancing the health and well-being of students.

### Study limitations

The study has several limitations. First, the intervention lasted only 5 weeks, which may not be sufficient to observe long-term and sustainable changes in PA behaviors. This short duration might affect the accuracy of the results, and longer-term studies are needed to assess the enduring effects of the intervention.

Moreover, the sample size was relatively small, which may restrict the generalizability of the findings to other populations. Future research should incorporate larger sample sizes to improve the precision and applicability of the results. The data collection relied on self-reported questionnaires, which could introduce response biases and affect the accuracy of the data. This potential limitation suggests that the results may not fully reflect the actual behavior of participants. Moreover, this research was done in a specific geographical area, which may limit the applicability of the results to different settings or populations. Replicating this research in diverse locations and among varied groups could provide more comprehensive insights into the effectiveness of the intervention.

## Conclusion

This study evaluates the impact of school-based peer education on improving physical activity (PA) among high school girls. The results suggest that the peer education intervention, based on the Theory of Planned Behavior (TPB), may significantly enhance PA levels while also promoting overall well-being benefits. Evidence regarding the effectiveness of school-based peer education on PA is limited, indicating a need to shift focus towards testing its applicability and effectiveness within school settings, such as by integrating this program into school curricula. Likewise, the current study explored that all constructs of TPB significantly predict the level of physical activity, and perceived behavioral control is the strongest predictor of PA behavior among the TPB constructs. Therefore, it is feasible to implement school-based peer education for adolescent girls based on promoting high school girls’ positive attitudes toward PA outcomes, enhancing their perceived ability to manage and engage in physical activity, and effectively utilizing appropriate subjective norms (such as the influence of parents, classmates, and teachers). It is also feasible to conduct this school-based peer education using a randomized control trial design based on longitudinal data in different sociodemographic factors to determine such intervention effectiveness. Additionally, this research can be a foundation for developing health policies and peer education guidelines in the Ministry of Education’s promotion.

## Data Availability

The original contributions presented in the study are included in the article/supplementary material, further inquiries can be directed to the corresponding author.
